# Adenine Nucleotide Translocase 1 Expression Is Coupled to the HSP27-Mediated TLR4 Signaling in Cardiomyocytes

**DOI:** 10.3390/cells8121588

**Published:** 2019-12-06

**Authors:** Julia Winter, Elke Hammer, Jacqueline Heger, Heinz-Peter Schultheiss, Ursula Rauch, Ulf Landmesser, Andrea Dörner

**Affiliations:** 1 Department of Cardiology, Charité—Universitätsmedizin Berlin, corporate member of Freie Universität Berlin, Humboldt-Universität zu Berlin, and Berlin Institute of Health, Campus Benjamin Franklin, 12200 Berlin, Germany; Julia.Winter@charite.de (J.W.); Ursula.Rauch@charite.de (U.R.); Ulf.Landmesser@charite.de (U.L.); 2 DZHK (German Centre for Cardiovascular Research, partner site Berlin), 10785 Berlin, Germany; 3 Institute of Chemistry and Biochemistry, Structural Biochemistry, Free University Berlin, 14195 Berlin, Germany; 4 Interfaculty Institute of Genetics and Functional Genomics, Ernst-Moritz-Arndt-University, 17475 Greifswald, Germany; Hammer@uni-greifswald.de; 5 German Centre for Cardiovascular Research (DZHK), partner site Greifswald, 17475 Greifswald, Germany; 6 Institute of Physiology, Justus-Liebig-University, 35392 Giessen, Germany; Jacqueline.Heger@physiologie.med.uni-giessen.de; 7 Institute of Cardiac Diagnostics and Therapy (IKDT), 12203 Berlin, Germany; Heinz-Peter.Schultheiss@charite.de

**Keywords:** adenine nucleotide translocase 1, heat shock protein 27, toll-like receptor 4, mitochondria, cell signaling, cardioprotection, myocardial infarction

## Abstract

The cardiac-specific overexpression of the adenine nucleotide translocase 1 (ANT1) has cardioprotective effects in various experimental heart disease models. Here, we analyzed the link between ANT1 expression and heat shock protein 27 (HSP27)-mediated toll-like receptor 4 (TLR4) signaling, which represents a novel communication pathway between mitochondria and the extracellular environment. The interaction between ANT1 and HSP27 was identified by co-immunoprecipitation from neonatal rat cardiomyocytes. ANT1 transgenic (ANT1-TG) cardiomyocytes demonstrated elevated HSP27 expression levels. Increased levels of HSP27 were released from the ANT1-TG cardiomyocytes under both normoxic and hypoxic conditions. Extracellular HSP27 stimulated TLR4 signaling via protein kinase B (AKT). The HSP27-mediated activation of the TLR4 pathway was more pronounced in ANT1-TG cardiomyocytes than in wild-type (WT) cardiomyocytes. HSP27-specific antibodies inhibited TLR4 activation and the expression of HSP27. Inhibition of the HSP27-mediated TLR4 signaling pathway with the TLR4 inhibitor oxidized 1-palmitoyl-2-arachidonoyl-sn-glycero-3-phosphocholine (OxPAPC) reduced the mitochondrial membrane potential (∆ψ_m_) and increased caspase 3/7 activity, which are both markers for cell stress. Conversely, treating cardiomyocytes with recombinant HSP27 protein stimulated TLR4 signaling, induced HSP27 and ANT1 expression, and stabilized the mitochondrial membrane potential. The activation of HSP27 signaling was verified in ischemic ANT1-TG heart tissue, where it correlated with ANT1 expression and the tightness of the inner mitochondrial membrane. Our study shows a new mechanism by which ANT1 is part of the cardioprotective HSP27-mediated TLR4 signaling.

## 1. Introduction

Adenine nucleotide translocase (ANT) plays a central role in the cellular energy supply, facilitating the transfer of intramitochondrial ATP and extramitochondrial ADP across the inner mitochondrial membrane [1]. It also regulates the opening of the mitochondrial permeability transition pore (MPTP), which ultimately mediates apoptotic and necrotic processes [2]. Mutations in the *ANT1* gene, which is the predominant ANT isoform in the heart, as well as reduced levels of ANT1 protein are related to complex human diseases that are accompanied by serious cardiac symptoms [3,4]. Knockout of *ANT1* results in hypertrophic cardiomyopathy and sudden cardiac death in mice [3]. In ischemic cardiomyopathy, *ANT1* gene expression is reduced, resulting in impaired mitochondrial ATP/ADP transportation [5]. Disturbed ANT1 function leads to insufficient energy supply, increased oxidative stress and cell death [6,7]. Thus, ANT1 is significantly involved in the pathophysiology of cardiac disease. 

On the other hand, heart-specific overexpression of ANT1 has distinctive cardioprotective effects in various experimental heart diseases, such as hypertension-induced hypertrophic cardiomyopathy, as well as diabetic or ischemic heart disease [8,9,10]. After myocardial infarction, ANT1 overexpression reduced the infarct size and prolonged survival [8]. Furthermore, ANT1 overexpression increased the activity of the respiratory chain complexes and attenuated reactive oxygen species (ROS) production and oxidative stress. It also stabilized mitochondrial membrane potential (∆ψ_m_) by blocking the opening of the MPTP [11]. Thus, ANT1 is an important component in anti-oxidative cell-protective processes. 

Additional studies indicated that ANT1 overexpression affects cell signaling by supporting the activity of protein kinase B (AKT); however, the underlying mechanism is not yet understood [12]. AKT activity is also positively affected by heat shock protein 27 (HSP27), a member of the small HSP family [13]. It mainly acts as a molecular chaperone and facilitates the refolding of partially denatured proteins into active conformations. In addition, HSP27 positively affects several ANT1-related processes. For instance, it has anti-apoptotic and anti-oxidative properties [14] and supports the activity of protective cell signaling components such as AKT [13]. HSP27 is located in the cytosol, nucleus, and mitochondria, where it performs protective functions [15,16,17]. In addition, HSP27 can be secreted and has several extracellular functions in modulating cellular, vascular, and immunological processes [18,19].

Here, we analyzed the interrelation between HSP27 and ANT1 and its function in the cell-protective processes, occurring in ANT1-overexpressing cardiomyocytes during hypoxia and in ischemic hearts.

## 2. Materials and Methods

### 2.1. Animals

Wild-type (WT) and heart-specific ANT1-TG Sprague-Dawley rats (TGRMHCrANT1 [ANT]) have been generated in our institute and described in detail by Walther et al. [9]. Rats were housed in Charité core facility for animal experiments (FEM) under standard conditions according to the international guidelines of Directive 2010/63/EU of the European Parliament. Studies were approved by the institutional animal care committee (G0158/08; T0449/08). Studies have therefore been performed in accordance with the ethical standards laid down in the 1964 Declaration of Helsinki and its later amendments.

### 2.2. Cardiomyocyte Treatment

Neonatal cardiomyocytes from age-matched, two- to three-day-old WT and ANT1-TG pups independent of sex were isolated and cultured as we previously described [12].

Cardiomyocytes were starved for 2 h in serum-free culture medium (low glucose DMEM supplemented with 1% penicillin/streptomycin, and 2 μM 5-fluoro-2′-deoxyuridine) before any experimental procedures. Hypoxic conditions were induced in a humidified, airtight, controlled atmosphere culture chamber (Modular Incubator Chamber; Billups-Rothenberg, Del Mar, CA, USA) placed in a standard 37 °C incubator. The chamber was flushed with pre-analyzed hypoxic gas (94% N_2_, 5% CO_2_, 1% O_2_) for 4 min at 25 L/min before sealing cells inside for 24 h. Normoxic cardiomyocytes were maintained at 37 °C in the same incubator with a normoxic atmosphere (74% N_2_; 5% CO_2_; 21% O_2_) for 24 h.

Culture medium from hypoxic ANT1-TG cardiomyocytes that contains released HSP27 was pre-incubated with HSP27-specific antibodies (10 µg/mL, Santa Cruz Biotechnology, Dallas, TX, USA) or non-specific goat IgG (10 µg/mL, Santa Cruz Biotechnology, Dallas, TX, USA) for 2 h in the dark. Normoxic WT and ANT1-TG cardiomyocytes were treated with the pre-conditioned medium for 30 min.

In addition, WT and ANT1-TG cardiomyocytes were treated with recombinant HSP27 (rHSP27) (endotoxin-free, 4 µg/mL, Enzo Life Sciences, Farmingdale, NY, USA) suspended in serum-free culture medium and cultivated for 30 min at 37 °C under normoxic conditions [19]. Thirty µg/mL oxidized phospholipid-1-palmitoyl-2-arachidonoyl-*sn*-glycerol-3-phosphatidylcholine (OxPAPC, InvivoGen, San Diego, CA, USA), a TLR4 inhibitor, was suspended in rHSP27-containing, serum-free medium and pre-incubated for 2 h in the dark before stimulation of cardiomyocytes for 30 min at 37 °C under normoxic conditions. Additionally, the AKT inhibitor triciribine (2 µM) was added to the culture medium for 24 h, as previously described [12]. All cardiomyocytes were immediately processed upon completion of treatment. Protein was stored at −20 °C until Western blot analyses were performed.

### 2.3. Collection of HSP27-Containing Culture Supernatant

Primary cardiomyocytes of WT and ANT1-TG neonatal rats were cultivated for 24 h under hypoxic conditions in a serum-free culture medium. Supernatant was collected and gently centrifuged at 1200× *g* (rotor JA 25-50, Beckmann Coulter, Fullerton, CA, USA) for 5 min. A second centrifugation was performed at 13,400× *g* (rotor FA-45-6-30, Eppendorf, Hamburg, Germany) at 4 °C for 30 min. The supernatant was used for Western blot analysis. For this, 500 µL supernatant samples were precipitated with 1500 µL ice-cold acetone overnight at −20 °C. Subsequently, the samples were centrifuged at 16,000× *g* for 30 min at 4 °C. The pellet was resuspended in denaturing sample buffer (1× NuPAGE sample buffer with 50 mM dithiothreitol, Life Technologies, Carlsbad, CA, USA), incubated for 10 min at 100 °C and analyzed by Western blotting.

### 2.4. Protein Isolation

Isolated cardiomyocytes or frozen tissue (see below) were lysed in ice-cold lysis buffer (Cell Signaling, Boston, MA, USA), containing PhosSTOP phosphatase inhibitors and a complete mini protease inhibitor (both from Roche Diagnostics Deutschland GmbH, Mannheim, Germany) as mentioned by the manufacturer. Lysates were centrifuged at 16,000× *g* for 30 min at 4 °C. Mitochondrial protein was isolated from heart tissue as described by Smith [20]. The cytosolic fraction resulting from the mitochondrial preparation was additionally centrifuged at 16,000× *g* for 90 min at 4 °C. Protein concentration was determined by bicinchoninic acid test (Pierce, Bonn, Germany).

### 2.5. Western Blot Analysis

Equal amounts of protein samples (25 µg) were separated on 4–12% polyacrylamide minigels (NuPAGE^®^ Novex Bis-Tris-Gels, Life Technologies, Carlsbad, CA, USA) and transferred onto a polyvinylidene fluoride membrane (BioRAD, Munich, Germany). Five to six samples of two groups (ANT1-TG_treated_ vs. WT_treated_, ANT1-TG_untreated_ vs. WT_untreated_, WT_untreated,_ vs. WT_treated_, and ANT1-TG_untreated_ vs. ANT1-TG_treated_) were applied to one minigel. Using corresponding samples, data from the different blots were equalized to each other. Western blots were performed using a standard protocol with specific primary antibodies against HSP27 (Santa Cruz Biotechnology, Dallas, TX, USA), TLR4 (BIOSS Antibodies, Woburn, MA, USA) ANT1 [21], AKT, phospho-AKT (Ser473), phospho-NFkB/p65 (Ser276), HSP60 (all from Cell Signaling, Boston, MA, USA), cytochrome C (CytC) and NFκB/p65 (Santa Cruz Biotechnology, Inc., Heidelberg, Germany), phospho-HSP27 (Ser85) (ThermoFisher Scientific, GmbH, Schwerte, Germany), and with HRP-conjugated goat anti-rabbit, goat anti-mouse or porcine anti-goat secondary antibodies (Dako, Glostrup, Denmark). Finally, membranes were stained with Coomassie blue, scanned, and the density of each lane was determined and used for normalization [22]. Bands between 130kDa and 170kDa are shown as a loading control in the graphs.

### 2.6. Immunoprecipitation and Mass Spectrometric Analysis

Immunoprecipitation was performed as described before [8]. For pre-cleaning, 100 µL protein A/G agarose beads (Pierce Protein Biology Products, IL, USA) were added to 100 µg lysed protein from normoxic and hypoxic WT or ANT1-TG cardiomyocytes, completed with fresh phosphatase and protease inhibitors. Samples were filled up to a final volume of 150 µL with IP-buffer (25mM Tris-HCl, 600 mM NaCl, pH 7.2) and incubated for 150 min under gentle shaking at room temperature. After centrifugation, the pre-cleaned supernatant was mixed with 5 µg ANT-specific antibody (Santa Cruz Biotechnology, Heidelberg, Germany) or unspecific IgGs (negative control) and incubated overnight under gentle shaking at 4 °C. The protein-antibody solution was added to 100 µL protein A/G agarose beads and incubated at 4 °C. The supernatant was discarded, and beads were washed 3 times with IP-buffer and denatured in sample buffer (Invitrogen, Carlsbad, CA, USA) containing 10 mM DTT for 10 min at 70 °C. After centrifugation, the supernatant was subjected to SDS gel electrophoresis and Western blotting with ANT1- and HSP27-specific antibodies as described above.

For mass spectrometric analyses, beads were washed three times with 100 µL ammonium bicarbonate (20 mM) before proteolytic digestion of proteins with trypsin at room temperature under rotation for 16 h. The reaction was stopped with a final concentration of 1% acetic acid. Resulting peptide extracts were purified as described by Hammer et al. [23] and analyzed by Liquid Chromatography Electrospray Ionization Tandem Mass Spectrometric (LC-ESI-MS/MS) with a LTQ-Orbitrap-Velos mass spectrometer [24]. Proteins were identified via automated SORCERER/SEQUEST search (Sorcerer built 4.04, Sage-N Research Inc., Milpitas, CA, USA.) against the Swissprot rat database. The obtained spectral count (SC) data were normalized for total SC per sample. Additionally, SC of unspecific bound proteins (unspecific IgGs) was subtracted. Data were divided by protein-specific amino acid length to obtain normalized spectral abundance factors (NSAF). ANT specific interaction was assumed when enrichment was ≥ 1.5 and *p* < 0.05 (n = 3–5). The ratio between HSP27 and ANT1 was determined. 

### 2.7. Mitochondrial Membrane Potential (∆ψ_m_)

The JC-1 dye accumulates in mitochondria in a potential-dependent manner and produces a fluorescence emission shift from green (530 nm) to red (590 nm). Treated and untreated cardiomyocytes were loaded with JC-1 (3 μmol/L) (Sigma, Taufkirchen, Germany) for 20 min at 37 °C and then washed for 20 min. The cells were excited at 490 nm and 525 nm, and the emitted fluorescence was measured at 530 and 590 nm, respectively, using a filter wheel. Mitochondrial depolarization, which occurs in response to hypoxic stress and causes pro-apoptotic and necrotic pathways, was monitored as a decrease in the 590/530 fluorescence intensity ratio.

### 2.8. Caspase-3/7 Activity

Caspase activity was accessed using the *Apo***-***ONE Homogeneous Caspase*-3/7 Assay kit (Promega, Madison, WI, USA). Cardiomyocytes grown in 96-well plates were treated with rHSP27, HSP27-specific antibodies or OxPAPC for 24 h under hypoxia. Immediately after treatment, cardiomyocytes were incubated with kit reagents. After 3–4 h of incubation at 20 °C, fluorescence was measured at an emission wavelength of 521 nm and an excitation wavelength of 499 nm using a microplate reader. Data were normalized to cell numbers as determined after staining with 0.1% trypan blue by microscopic counting.

### 2.9. Myocardial Infarction

Myocardial infarction was induced in male WT and heart-specific ANT1-TG Sprague-Dawley rats (4–6 months old) by permanent ligation of the left descending coronary artery (LAD) during a previous study [8]. Infarcted heart tissue was dissected after 24 h of infarction and immediately frozen in liquid nitrogen, and stored at −80 °C. Frozen heart tissue of sham-operated WT (n = 5) and ANT1-TG (n = 6) and infarcted rats (WT, n = 6; ANT1-TG, n = 5) was used for protein extraction and Western blots as described above.

### 2.10. Correlation between ANT and HSP27 Transcription Levels in Human Heart Tissue

Data from microarray assays were generated from the left ventricular heart tissue of normal heart donors and patients with ischemic heart disease who underwent heart transplantation. Data were published in the NCBI Gene Expression Omnibus (GEO) database under the accession number GDS651, entitled “Heart failure arising from different etiologies”, and were analyzed. The expression levels of *HSPB1 (HSP27)* (https://www.ncbi.nlm.nih.gov/geo/tools/profileGraph.cgi?ID=GDS651:201841_s_at) in both controls and patients with ischemic cardiomyopathy were correlated with the transcription levels of *SLC25A4 (ANT1)* (https://www.ncbi.nlm.nih.gov/geo/tools/profileGraph.cgi?ID=GDS651:202825_at). 

### 2.11. Data Presentation and Statistical Analysis

Data are presented relative to values from the designated controls. Statistical analyses of more than two groups were performed using the two-way analysis of variance (ANOVA) test with Bonferroni as a post hoc test. Data are presented as the mean ± standard error of the mean (SEM). The mean values of the respective groups were correlated with each other, and Pearson’s correlation coefficient and a two-tailed *p*-value were calculated. Differences were considered to be significant at *p* < 0.05. Statistical analyses were performed using GraphPad Prism 6.07 (GraphPad Software, San Diego, CA, USA).

## 3. Results

### 3.1. ANT1 Overexpression Induces Increased HSP27 Expression

**Figure 1:**
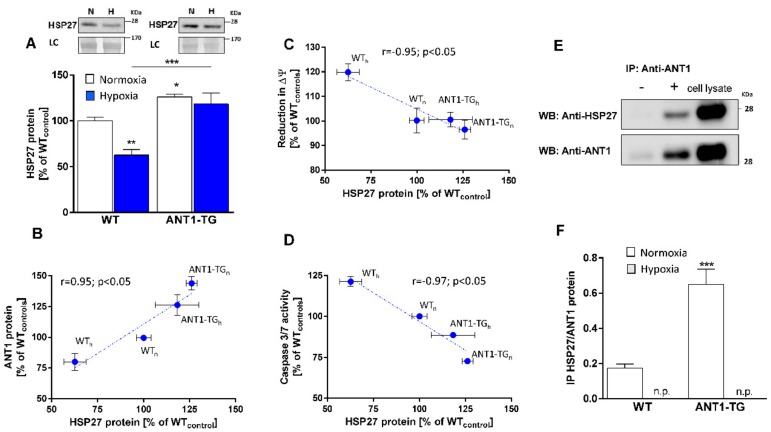
The interrelation between ANT1 and HSP27. (**A**) The upper panel shows representative Western blots for HSP27 with loading controls (LC) in normoxic (N) and hypoxic (H) WT and ANT1-TG cardiomyocytes. The lower panel shows the quantification of HSP27 protein levels in normoxic (**white** bars) and hypoxic (**blue** bars) WT and ANT1-TG cardiomyocytes (n = 4–5). The protein level in normoxic WT cardiomyocytes was set to 100% (WT_control_). The mean ± SEM values for (**B**) ANT1 protein level, (**C**) reduction in the mitochondrial membrane potential (∆ψ_m_), and (**D**) caspase-3/7 activity in normoxic (n) and hypoxic (h) WT and ANT1-TG cardiomyocytes were correlated with the mean HSP27 protein levels. Panel (**E**) shows representative Western blots for HSP27 and ANT1 from immunoprecipitates with (+) and without (-) ANT-specific antibodies. Total cell lysate was used as the control. (**F**) The ratio for immunoprecipitated HSP27/ANT1 was determined for normoxic (white bars) and hypoxic WT and ANT1-TG cardiomyocytes (n = 3–5). HSP27 was not present (n.p.) in the precipitates of hypoxic cardiomyocytes. * *p* < 0.05; ** *p* < 0.01; *** *p* < 0.001 vs. WT_control_ or vs. indicated conditions.

We previously demonstrated that ANT1 overexpression resulted in the attenuation of cell death under hypoxic conditions, as demonstrated by reductions in cellular lactate dehydrogenase release, caspase 3 activity, and DNA fragmentation, and the stabilization of the mitochondrial membrane potential (∆ψ_m_) [12]. Heat shock proteins, such as HSP27, are important components that protect the cell from damage. We observed that normoxic ANT1-TG cardiomyocytes expressed higher levels of HSP27 protein than WT cardiomyocytes (Figure 1A). While HSP27 expression levels remained stable in hypoxic ANT1-TG cardiomyocytes, they decreased in hypoxic WT cardiomyocytes. The tight correlation between HSP27 and ANT1 protein levels implies interaction of both proteins (Figure 1B, Supplementary Figure S1G). In addition, the increase in the HSP27 protein expression correlated with reduced depolarization of the mitochondrial membrane and reduced caspase-3/7 activity (Figure 1C,D), suggesting that HSP27 plays a significant role in mitochondrial function and cellular stability.

### 3.2. HSP27 Interacts with ANT1 Protein

To determine whether an interaction occurred between ANT1 and HSP27, immunoprecipitation (IP) from cardiomyocyte protein extracts was performed, using an ANT-specific antibody. HSP27 co-immunoprecipitated with the ANT1 protein (Figure 1E) from normoxic cardiomyocytes. A higher amount of HSP27 protein was precipitated from ANT1-TG cell lysates than from WT lysates under normoxic conditions determined by LC-ESI-MS/MS (Figure 1F). In contrast, no HSP27 protein was detected in the precipitates from WT or ANT1-TG cardiomyocytes under hypoxic conditions. Thus, there is an interaction between HSP27 and ANT1 protein in normoxic cardiomyocytes, which is disintegrated under hypoxic conditions.

### 3.3. ANT1 Overexpression Stimulates HSP27 Release and TLR4 Expression

**Figure 2:**
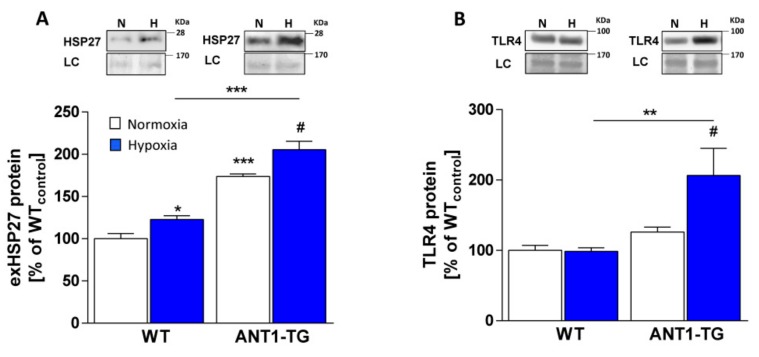
ANT1 overexpression resulted in elevated cellular HSP27 release and TLR4 protein amount. HSP27 abundance was determined in the culture medium, and the TLR4 expression levels are presented for normoxic (**white** bars) and hypoxic (**blue** bars) WT and ANT1-TG cardiomyocytes. The upper panels show representative Western blots for (**A**) extracellular HSP27 (exHSP27) and (**B**) TLR4 with loading control (LC) for normoxic (N) and hypoxic (H) WT and ANT1-TG cardiomyocytes. The graphs present the protein quantifications of (A) exHSP27 and (B) TLR4 levels, shown as the relative percentage compared with the levels in normoxic WT cardiomyocytes (WT_control_). n = 5–6; * *p* < 0.05, ** *p* < 0.01, *** *p* < 0.001 vs. WT_control_ or as indicated; ^#^ *p* < 0.05 vs. normoxic ANT1-TG.

ANT1 overexpression resulted not only in increased HSP27 expression levels but also in the increased release of HSP27 protein (Figure 2A) from normoxic cardiomyocytes. Hypoxia further elevated the levels of extracellular HSP27 (exHSP27) released from both WT and ANT1-TG cardiomyocytes. However, significantly higher levels of HSP27 were set free from ANT1-TG cardiomyocytes than from WT cardiomyocytes.

Furthermore, exHSP27 has been shown to react with toll-like receptor 4 (TLR4) [18]. Normoxic ANT1-TG cardiomyocytes displayed a slight, but not significant, elevation in TLR4 levels (26.1 ± 15.6%; *p* = 0.0556) (Figure 2B). TLR4 protein was significantly increased under hypoxic conditions in ANT1-TG cardiomyocytes but not in WT cardiomyocytes. This implies that ANT1-TG cardiomyocytes increase the release of HSP27 to activate TLR4.

### 3.4. Inhibition of exHSP27, Using HSP27-Specific Antibodies, Decreases TLR4 and HSP27 Expression Levels

**Figure 3:**
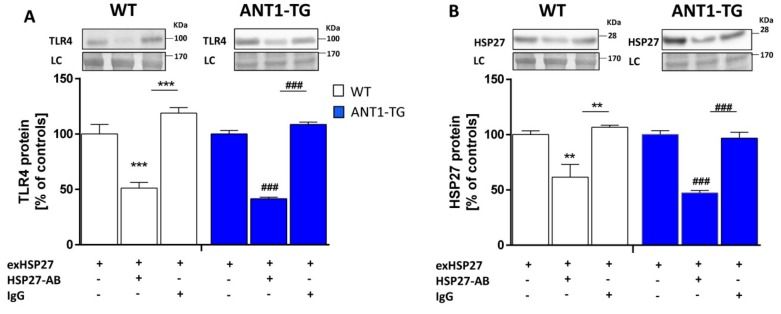
Inhibition of extracellular HSP27 reduced HSP27 expression and TLR4 activation. WT (**white** bars) and ANT1-TG cardiomyocytes (**blue** bars) were treated with the cultured supernatant from hypoxic ANT1-TG cardiomyocytes, which contained the highest concentration of secreted HSP27 (exHSP27). Treatment was performed in the presence or absence of either HSP27-specific antibodies or non-specific IgGs. The upper panels show representative Western blots for (**A**) TLR4 and (**B**) HSP27, with a loading control (LC). The quantification of TLR4 and HSP27 levels is shown in the lower panels. Data were calculated as relative percentages of the levels in antibody-free WT and ANT1-TG controls. n = 4–5 ** *p* < 0.01, *** *p* < 0.001 vs. WT controls or as indicated. ^###^ *p* < 0.001 vs. ANT1-TG controls or as indicated.

To demonstrate the impact of native exHSP27 on TLR4 expression, we co-cultured normoxic WT and ANT1-TG cardiomyocytes in the presence or absence of either an HSP27-specific antibody or non-specific IgGs, as the control. HSP27 antibody treatment resulted in decreased TLR4 expression levels (Figure 3A) in both WT and ANT1-TG cardiomyocytes, whereas non-specific IgGs had no effects on the TLR4 expression. Besides, treatment with HSP27-specific antibodies decreased HSP27 expression levels in both WT and ANT1-TG cardiomyocytes, whereas non-specific IgGs did not influence the cellular HSP27 protein levels (Figure 3B). Thus, there is a cross-talk between exHsp27 and intracellular HSP27 expression that appears to be mediated by TRL4.

### 3.5. ANT1-TG Cardiomyocytes Are More Responsive than WT Cardiomyocytes to HSP27-Mediated TLR4 Signaling, Which Upregulated HSP27 and ANT1 Expression

**Figure 4:**
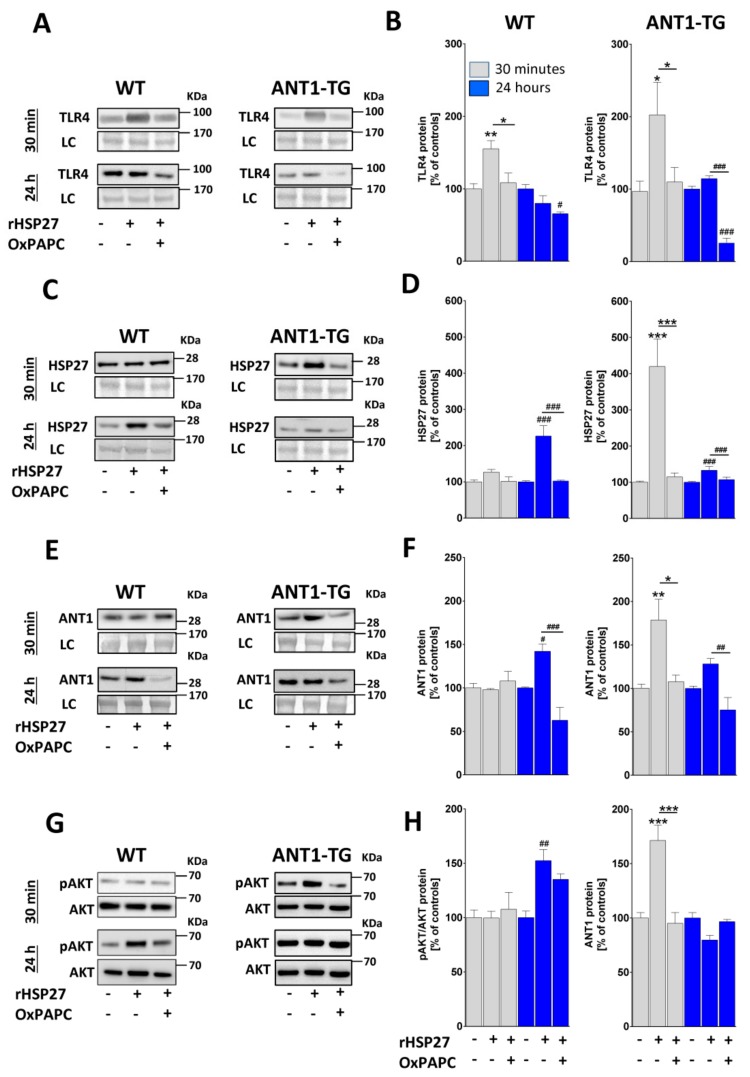
Inhibition of TLR4 diminished HSP27-induced HSP27 and ANT1 expression and AKT activation. Normoxic WT and ANT1-TG cardiomyocytes were treated with recombinant HSP27 (rHSP27) in the presence or absence of a TLR4 inhibitor (OxPAPC) for 30 min (**gray** bars) and 24 h (**blue** bars). Representative Western blots for (**A**) TLR4, (**C**) HSP27, (**E**) ANT1, and (**G**) pAKT/AKT, with a corresponding loading control (LC), are presented on the left side. The relative quantities of (**B**) TLR4, (**D**) HSP27, (**F**) ANT1, and (**H**) pAKT/AKT were calculated as percentages of the levels in the corresponding untreated WT and ANT1-TG controls and are presented as graphs. n = 5–6, * *p <* 0.05, ** *p* < 0.01, *** *p* < 0.001 vs. 30-min controls or as indicated, or ^#^ *p* < 0.05, ^##^ *p* < 0.01, ^###^ *p* < 0.001 vs. 24-h controls or as indicated.

Normoxic WT and ANT1-TG cardiomyocytes were treated with recombinant HSP27 protein (rHSP27) for 30 min (short-term treatment) or 24 h (long-term treatment). Short-term treatment caused the rapid elevation of TLR4 protein levels (Figure 4A,B), which subsequently normalized to basal levels in both WT and ANT1-TG cardiomyocytes after 24 h. The short-term treatment of ANT1-TG cells with rHSP27 induced a strong increase in HSP27 expression, which decreased over time. In contrast, the short-term treatment of WT cardiomyocytes with rHSP27 resulted in an insignificant rise in HSP27 expression (26.6 ± 18.6%); however, HSP27 expression significantly increased in WT cardiomyocytes following long-term treatment with rHSP27 (Figure 4C,D).

ANT1-TG cardiomyocytes, but not WT cardiomyocytes, displayed elevated ANT1 protein levels following short-term rHSP27 treatment, which remained slightly increased after the 24-h treatment with rHSP27 (Figure 4E,F). WT cardiomyocytes displayed elevated ANT1 expression levels only after long-term treatment with rHSP27. 

The simultaneous treatment of cardiomyocytes with both rHSP27 and the TLR4 inhibitor OxPAPC completely suppressed all of the effects observed with rHSP27 treatment alone, in both WT and ANT1-TG cardiomyocytes (Figure 4A–H). These results show that HSP27 signals by TLR4 and induces an increase in HSP27 and ANT1 protein expression. ANT1-TG cardiomyocytes reacted significantly faster to HSP27-mediated TLR4 signaling than WT cardiomyocytes. 

### 3.6. ANT1-TG Cardiomyocytes Respond to HSP27 Treatment with TLR4-Dependent AKT Activation

HSP27 stabilizes the activity of the cell-protective AKT, which is a downstream target of TLR4 signaling [13]. AKT has been demonstrated to be highly activated in hypoxic ANT1-TG cardiomyocytes [12]. HSP27 treatment for 30 min resulted in increased AKT phosphorylation in ANT1-TG cardiomyocytes, whereas WT showed an increase only after 24 h (Figure 4G and H). The simultaneous treatment of cardiomyocytes with both rHSP27 and OxPAPC blocked AKT phosphorylation in ANT1-TG cardiomyocytes, whereas WT cardiomyocytes did not react to the presence of OxPAPC. Hence, the HSP27-mediated activation of AKT, which is sensitive to OxPAPC, is especially pronounced in ANT1-TG cardiomyocytes.

### 3.7. HSP27 and ANT1 Expression Were AKT Activity-Dependent in ANT1-TG Cardiomyocytes

**Figure 5:**
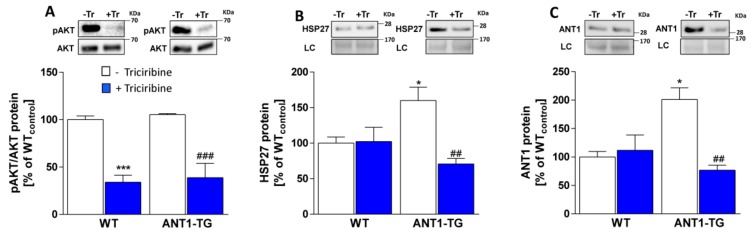
Inhibition of AKT reduced HSP27 and ANT1 expression levels in ANT1-TG, but not WT, cardiomyocytes. WT and ANT1-TG cardiomyocytes were cultured for 24 h in the presence (**blue** bars) or absence of triciribine (Tr) (**white** bars). The upper panels show representative Western blots for (**A**) pAKT and pan-AKT, (**B**) HSP27, and (**C**) ANT1, with a loading control (LC), in WT and ANT1-TG cardiomyocytes. The lower panels show the ratios for pAKT/AKT and the quantifications of HSP27 and ANT1 protein levels. The levels in untreated WT cardiomyocytes were set to 100% (WT_control_). n = 5–6, * *p* < 0.05, *** *p* < 0.001 vs. WT controls or as indicated. ^##^ *p* < 0.01, ^###^ *p* < 0.01 vs. ANT1-TG controls.

To demonstrate the effects of AKT activity on HSP27 and ANT1 expression levels, WT and ANT1-TG cardiomyocytes were treated with the AKT inhibitor triciribine. Triciribine significantly reduced AKT phosphorylation in both WT and ANT1-TG cardiomyocytes (Figure 5A).

Intracellular HSP27 levels declined after triciribine treatment in ANT1-TG cardiomyocytes, but not in WT cardiomyocytes (Figure 5B). ANT1 protein levels were not affected by triciribine treatment in WT cardiomyocytes, whereas ANT1-TG cardiomyocytes exhibited reduced ANT1 protein levels following AKT inhibition (Figure 5C). Consequently, AKT activity is essential for HSP27 and ANT1 expression in ANT1-TG cardiomyocytes.

### 3.8. HSP27-Induced TLR4 Activation Increases ∆ψ_m_ and Suppresses Caspase-3/7 Activity

**Figure 6:**
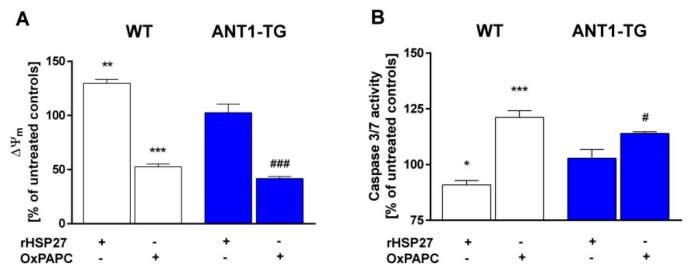
Inhibition of the HSP27-mediated TLR4 pathway reduced ∆ψ_m_ and increased caspase 3/7 activity. WT (**white** bars) and ANT1-TG cardiomyocytes (**blue** bars) were cultured under hypoxic conditions for 24 h in the absence or presence of rHSP27 and OxPAPC. (**A**) ∆ψ_m_ and (**B**) caspase-3/7 activity were determined for the cardiomyocytes, and the data were calculated as relative percentages of the levels in the corresponding untreated WT and ANT1-TG controls. n = 3–4, * *p* < 0.05, ** *p* < 0.01, *** *p* < 0.001 vs. WT controls and ^#^ *p* < 0.05, ^###^ *p* < 0.001 vs. ANT1-TG controls.

It was analyzed whether rHSP27 treatments and the inhibition of TLR4 signaling by OxPAPC have effects on ∆ψ_m_, which is regulated by ANT1 [25]. Stimulation with rHSP27 increased ∆ψ_m_ in hypoxia stressed WT cardiomyocytes, but had no effects in ANT1-TG cells (Figure 6A). 

However, OxPAPC treatment reduced ∆ψ_m_ in both WT and ANT1-TG cardiomyocytes. Caspase-3/7 activity, another marker for cellular stress, was reduced by rHSP27 treatment in WT cardiomyocytes, but not in ANT1-TG cardiomyocytes (Figure 6B). However, treating cardiomyocytes with OxPAPC resulted in increased caspase-3/7 activity, for both cell types. Thus, inhibition of TLR4 signaling by OxPAPC abolishes the cell-protective effect of ANT1 overexpression, showing the significance of this pathway for the protective processes observed in ANT1-TG cardiomyocytes.

### 3.9. ANT1 Overexpression Increases HSP27 Signaling in Infarcted Hearts that Correlates with Mitochondrial Stability

ANT1 overexpression has been shown to be cardioprotective in rats with acute myocardial infarction [8]. Cardioprotection was based on increased mitochondrial intactness and reduced oxidative stress, which increased the survival rate by 57% and reduced infarct size by 26%.

**Figure 7:**
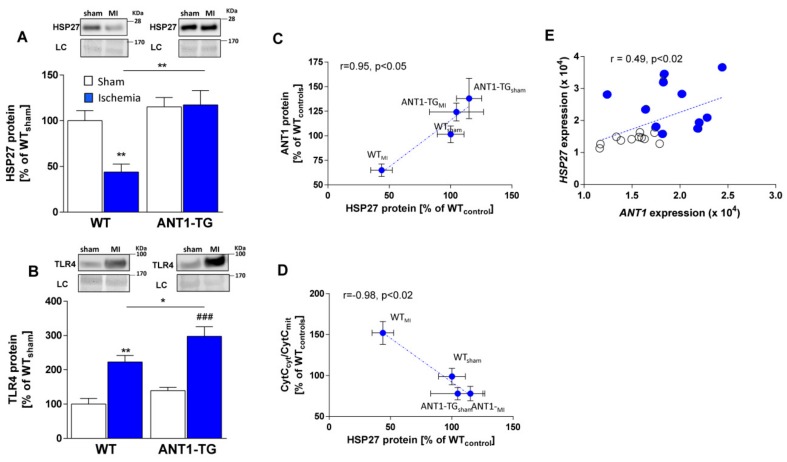
ANT1 overexpression stimulated the HSP27-mediated TLR4 pathway in infarcted hearts, which was connected with increased mitochondrial integrity. The upper panels show representative Western blots for (**A**) HSP27 and (**B**) TLR4, with a loading control (LC). The quantification of HSP27 and TLR4 protein levels in heart tissue from WT and ANT1-TG sham-operated (**white** bars) and ischemic (MI, **black** bars) animals are shown in the lower graphs. The mean HSP27 levels from sham-treated and infarcted WT and ANT1-TG hearts were correlated with the mean (**C**) ANT1 protein levels and the mean (**D**) CytC_cyt_/CytC_mit_ ratios. Pearson’s correlation coefficient and a two-tailed *p*-value were calculated. Data are presented as percentages of the values in sham-treated WT hearts (WT_control_). n = 5–6; * *p* < 0.05; ** *p* < 0.01 vs. WT controls and ^###^ *p* < 0.001 vs. ANT1-TG controls. (**E**) The transcript levels of *HSP27* and *ANT1* were correlated in explanted left ventricular heart tissue from heart donors (filled circles) and patients with ischemic cardiomyopathy (open circles). Data were taken from the NCBI, GEO database, Accession number: GDS651, n = 22.

HSP27 expression levels were consistent between sham-operated WT and ANT1-TG hearts (Figure 7A). However, HSP27 protein levels decreased in infarcted WT hearts but remained stable in infarcted ANT1-TG hearts. The myocardial TLR4 protein level increased under ischemic conditions in both WT and ANT1-TG hearts, although TLR4 protein levels were significantly higher in ANT1-TG hearts than in WT hearts (Figure 7B).

As observed in cardiomyocytes, HSP27 expression levels positively correlated with ANT1 expression levels (Figure 7C) in infarcted heart tissue. In addition, HSP27 expression levels were negatively correlated with the cytosolic to mitochondrial cytochrome C ratio (CytC_cyt_/CytC_mit_), an indicator of intact inner mitochondrial membranes (Figure 7D). Thus, the HSP27-mediated TLR4 pathway contributes to the reduction of permeability of the inner mitochondrial membrane and cardioprotection in ANT1-TG hearts.

### 3.10. ANT1 and HSP27 Transcription Levels Are Correlated in Explanted Hearts from Donors and Patients with Ischemic Cardiomyopathy

To test the potential relevance of the interrelation between HSP27 and ANT1 levels in infarcted human hearts, we used microarray assay data that is publicly available in the NCBI GEO database, under the accession number GDS651. Data were generated from the left ventricular heart tissue of normal donors and patients with ischemic cardiomyopathy. mRNA levels of HSP27 (*HSPB1*) correlated with those of ANT1 (*SLC25A4*) mRNA (Figure 7E). This implies that HSP27-mediated ANT1 expression may be clinically relevant for ischemic cardiomyopathy.

## 4. Discussion

**Figure 8:**
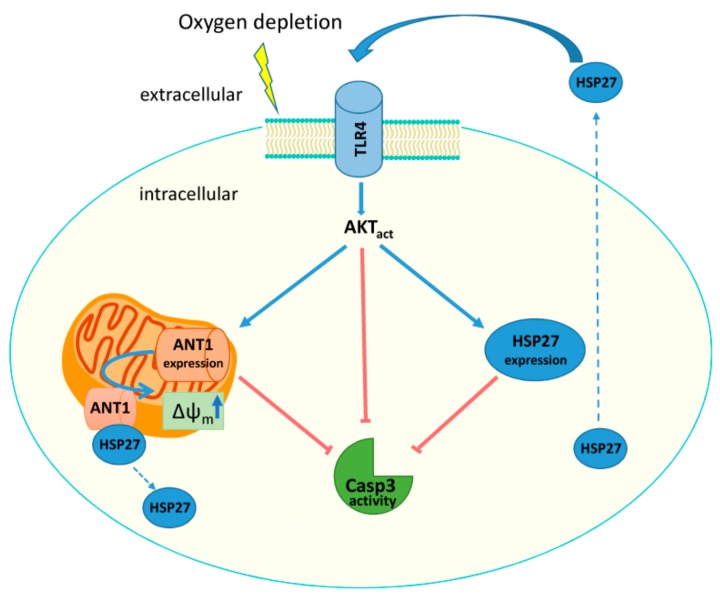
ANT1-TG cardiomyocytes are highly sensitive to the TLR4-mediated AKT pathway activated by exHSP27. HSP27 is a component of the ANT1-specific protein-protein network, which disappears during hypoxia. ANT1-TG cardiomyocytes react to hypoxia with an increased release of HSP27 protein. This extracellular HSP27 activates TLR4 and downstream AKT signaling. The HSP27-mediated TLR4 signaling subsequently induces HSP27 and ANT1 expression that support the stabilization of ∆ψ_m_ and the inhibition of caspase-3/7 activity, thereby leading to cell protection. Blue arrows indicate activation/increase, red lines indicate inhibition, and dashed arrows indicate release.

This study demonstrates a novel mechanism, in which ANT1 is part of the cardioprotective HSP27-mediated TLR4 signaling, shown in Figure 8. This pathway links mitochondria to the extracellular environment. HSP27 is a chaperone that stabilizes denatured proteins, modulates apoptosis, reduces oxidative stress, and serves as a signaling molecule [26]. We have revealed that HSP27 interacts with the ANT1 under normoxic conditions. Hypoxia disrupts the interaction between ANT1 and HSP27 and increases the secretion of HSP27 from the cell. Secreted HSP27 activates TLR4, which regulates the expression of ANT1 and HSP27 via AKT. Increased ANT1 and HSP27 expression levels enhance the stabilization of the mitochondrial membrane potential and inhibit caspase-3/7 activity, which consequently leads to cellular protection. ANT1 overexpression increases the responsiveness to extracellular HSP27. Thus, elevated ANT1 expression affects not only mitochondrial function but also intra- and extracellular signaling within heart tissue. This represents a novel communication pathway between mitochondria and the extracellular environment.

### 4.1. HSP27 Cooperates with ANT1

Here, we demonstrate that intracellular HSP27 levels are correlated with ANT1 protein levels in isolated rat cardiomyocytes, as well as in heart tissue from both rats and humans, indicating a close interaction between HSP27 and ANT1. Additionally, the co-precipitation of HSP27 and ANT1 corroborates this interaction. The overexpression of ANT1 increases the recruitment of HSP27 to the ANT1-specific protein-protein network (PPI). However, HSP27 does not bind to ANT1 under hypoxic conditions. HSP27 has been observed in the cytosol and organelles such as mitochondria and the nucleus [15,16,17]. Under stress conditions, HSP27 translocates within the cell and binds to various proteins that inhibit apoptosis [15,16,26]. Therefore, we argue that HSP27 molecules detach from the ANT1-specific PPI under hypoxia and act as mitochondrial signaling proteins that counteract cell stress. Indeed, HSP27 and ANT1 protein levels were positively correlated with ∆ψ_m_ and negatively correlated with caspase-3/7 activity, indicating that HSP27 and ANT1 reduce cell stress. Furthermore, ANT1 overexpression has been shown to stabilize the ∆ψ_m_ [11,12]. The collapse of ∆ψ_m_ causes the release of mitochondrial cytochrome c, an activator of the apoptosome that induces caspase-3 activity. HSP27 interacts with cytochrome c and caspase 9, blocking the activation of pro-caspase-3 [26]. Therefore, both ANT1 and HSP27 are responsible for the suppression of caspase-3 activity and make a common regulation reasonable.

### 4.2. ANT1 Overexpression Supports HSP27 Release, Which Increases Its Own Intracellular Expression

HSP27 can be secreted from both WT and ANT1-TG cardiomyocytes. Botulin et al. provided a comprehensive overview of the mechanisms underlying active HSP27 secretion [18]. Secreted HSP27 serves as a signaling molecule outside of the cell, binding to surface receptors on distant cellular targets to initiate outside-in signaling. The inhibition of secreted HSP27 using HSP27-specific antibodies reduces intracellular HSP27 expression levels in WT and ANT1-TG cardiomyocytes, while stimulation with rHSP27 induces it. Consequently, a positive feedback interaction exists between extracellular HSP27 secretion and intracellular HSP27 expression. This interaction is significantly stronger and faster in ANT1-TG cells, which may explain the increased stability of intracellular HSP27 levels in hypoxic ANT1-TG cardiomyocytes compared with WT cardiomyocytes.

### 4.3. HSP27 and ANT1 Synthesis are Commonly Regulated by TLR4-Mediated Signaling

ExHSP27 has been reported as being involved in various functions, including “outside-in” signaling, modulating cellular proliferation and immune responses, where it is thought to interact with TLRs [19,27]. TLR4 is the most prominent TLR isoform in the heart [28]. In the present study, we demonstrated that rHSP27 induces intracellular signaling via TLR4 activation, resulting in increased intracellular HSP27 levels. This pathway is linked to ANT1 expression and is thus much more pronounced in ANT1-TG cardiomyocytes. The correlation between ANT1 and HSP27 protein levels indicates the existence of a common TLR4-regulated pathway for ANT1 and HSP27 synthesis, which can be inhibited by HSP27-specific antibodies or a TLR4 inhibitor. The addition of rHSP27 reduced the depolarization of ∆ψ_m_ and activation of caspase 3/7, which usually occurs in response to hypoxia, coupling exHSP27 with mitochondrial function. ANT1-TG cardiomyocytes released larger amounts of HSP27 under hypoxic conditions, which appears to be sufficient for cell protection as adding further rHSP27 protein to the media had no effect on either ∆ψ_m_ or caspase-3/7 activity in ANT1-TG cardiomyocytes. This hypothesis is supported by the fact that ANT1-TG cardiomyocytes showed less caspase-3/7 activity under hypoxic conditions than WT cardiomyocytes. Thus, HSP27-mediated TLR4 signaling regulates ANT1 and HSP27 expression, as well as mitochondrial integrity, and supports cell-protective process in hypoxic ANT1-TG cardiomyocytes. 

We found that ANT1-TG cardiomyocytes were also more sensitive to HSP27 phosphorylation (Supplementary Figure S1). HSP27 phosphorylation, which supports HSP27′s survival-promoting activity [29], has been shown to affect ANT1 expression in neural cells [30,31], but this phenomenon was not observed in WT or ANT1-TG cardiomyocytes (for further explanation see File S1).

### 4.4. HSP27 Supports TLR4-Mediated AKT Signaling in ANT1-TG Cardiomyocytes

As demonstrated in a previous study, ANT1-TG cardiomyocytes respond to hypoxia with significantly increased AKT phosphorylation compared with WT cardiomyocytes [12]. In the present study, we revealed that ANT1-TG cardiomyocytes rapidly activated AKT in response to rHSP27-stimulation. This activation was blocked by the TLR4 inhibitor OxPAPC, indicating that this signaling cascade was initiated by TLR4 activation. AKT supports cell survival processes by blocking caspase-3 activity [32]. HSP27 has been shown to interact with AKT and stabilizes its activity [33]. Moreover, silencing TLR4-mediated AKT activity with OxPAPC resulted in reduced ∆ψ_m_ and elevated caspase 3/7 activity in hypoxic WT and ANT1-TG cardiomyocytes. In addition, both OxPAPC and the AKT inhibitor triciribine reduced HSP27 and ANT1 expression in ANT1-TG cardiomyocytes. We have previously demonstrated that the inhibition of AKT activity by triciribine decreases ∆ψ_m_ and removes the stabilizing effect of ANT1 overexpression on ∆ψ_m_ [12]. Consequently, AKT is pivotal during the regulation of cellular integrity in response to TLR4 stimulation by HSP27 in ANT1-TG cardiomyocytes. WT cardiomyocytes exhibited a delayed reaction to rHSP27 and did not reduce HSP27 and ANT1 expression following triciribine treatment. This result could be explained by a lower release of HSP27 (Figure 2A), which results in a different extracellular distribution of TLR4 ligands. The ratio of anti-apoptotic HSP27 to pro-apoptotic HSP60 [34], another TLR4 ligand, is significantly lower in WT cardiomyocytes (see Supplementary Figure S2B). Thus, HSP60, may occupy TLR4 and interfere with HSP27 binding, which could explain the reduced sensitivity of WT cardiomyocytes to HSP27. Stress like hypoxia even increased the release of HSP60 (Figure S2A) in WT cardiomyocytes and further decreased the proportion of cell-protective HSP27 to HSP60. In contrast, ANT1-TG cardiomyocytes did not increase the release of HSP60 under hypoxia. Thus, a lower ratio of extracellular HSP27/HSP60 may explain the lower reactivity of WT in comparison to ANT1-TG cardiomyocytes. 

### 4.5. HSP27-Mediated TLR4 Signaling Supports Cardioprotection

We determined that the HSP27-mediated TLR4 pathway is active in ischemic ANT1-TG rat hearts. Compared to infarcted WT hearts, the amount of myocardial HSP27 was significantly higher in infarcted ANT1-TG heart tissue and correlated with ANT1 expression and the stability of the mitochondrial inner membrane. This increase in myocardial HSP27 levels was accompanied by cardioprotection, as infarcted ANT1-TG rats have previously been shown to exhibit reduced infarct areas and increased survival rates in response to myocardial infarction [8]. The co-expression of ANT1 and HSP27 was also observed in left ventricular heart tissue from heart donors and patients with ischemic cardiomyopathy, demonstrating that this pathway may be of clinical relevance. Indeed, increased levels of Hsp27 or reduced levels of HSP27-specific autoantibodies have been correlated with a milder progression of the acute coronary syndrome in humans [35,36,37].

To date, the role played by TLR4 in the ischemic heart has remained inconclusive [38]. While the TLR4 knockout was shown to be cardioprotective in ischemic hearts, the activation of TLR4 by a sub-lethal dose of the TLR4 ligand lipopolysaccharide reduced the myocardial infarct area and improved cardiac functions in rats and rabbits [39,40]. In the present study, we observed that increased TLR4 activation by HSP27, coupled with increased HSP27 and ANT1 protein expression, is linked to cardioprotection. Thus, the contradictory observations regarding TLR4 in ischemic heart disease are most likely related to its different ligands (e.g., HSP27 and Hsp60), which are dispersed throughout ischemic hearts, where they interact with TLR4 and cause diverse outcomes. HSP27 protein is strongly correlated with ANT1 and mitochondrial integrity in heart tissue. Consequently, we demonstrated that the stabilization of mitochondrial function by ANT1 overexpression is linked to intra- and extracellular HSP27 signaling, thereby supporting the existence of a cell-protective TLR4 signaling pathway in ANT1-TG ischemic hearts.

## 5. Conclusions

In summary, HSP27 represents a component of the ANT1-specific PPI that disassociates from ANT1 under hypoxic conditions and may act as a mitochondrial signaling factor. ANT1-overexpressing cardiomyocytes intensify the expression and secretion of the HSP27 protein under hypoxic conditions. In turn, secreted HSP27 stimulates TLR4-dependent AKT activation and induces its HSP27’s own expression, as well as that of ANT1. In addition, HSP27-mediated signaling stabilizes the mitochondrial membrane potential and the inhibition of caspase-3/7, which leads to cellular protection. ANT1 overexpression supports the described mechanism in infarcted hearts. These findings may be of clinical relevance because ANT1 and HSP27 expression levels are correlated in patients with ischemic cardiomyopathy. Thus, changes in ANT1 expression levels affect intra- and extracellular HSP27 signaling, and HSP27 appears to be a potent stabilizer of ANT1 expression and, consequently, of the mitochondrial function.

## Supplementary Materials

The following figures are available online at https://www.mdpi.com/2073-4409/8/12/1588/s1, Figure S1: Phosphorylation of HSP27 and NFκB, Figure S2: ANT1 overexpression reduces the release of HSP60 under hypoxia.
