# Supplementary material for: Adenine Nucleotide Translocase 1 Expression Is Coupled to the HSP27-Mediated TLR4 Signaling in Cardiomyocytes
Source: Cells. 2019 Dec 6;8(12):1588. doi: 10.3390/cells8121588 (PMC6952976; doi:10.3390/cells8121588)
Supplement: Supplementary file 1 [file cells-08-01588-s001.zip › Supplemented files/Revised Suppl 1.docx]

*S1.1 ANT1 overexpression influences the phosphorylation of HSP27 and NFκB.*

HSP27 phosphorylation supports HSP27´s survival-promoting activity [29]. We found that ANT1-TG cardiomyocytes are more sensitive to HSP27 phosphorylation than WT cardiomyocytes (Figure S1A-C). Phosphorylated HSP27 has been shown to inhibit the inhibitor of kappa B kinase (IKK) activity [30] and consequently blocks the activation of the nuclear factor 'kappa-light-chain-enhancer' of activated B-cells (NFκB), a transcription factor that binds to the ANT1 promoter and suppresses ANT1 transcription in neural cells [31]. Indeed, we found that the increase in the percentage of phosphorylated HSP27 was negatively associated with the proportion of phosphorylated and thus activated NFκB (Figure S1D-F). However, HSP27 and NFκB phosphorylation did not correlate with the ANT1 expression (Figure S1G). These findings lead to the conclusion that the expression of ANT1 depends on the amount of HSP27 protein, but not on the HSP27 phosphorylation status. Besides, the NFκB-related regulation of ANT1 appears to be specific for neural cells and another yet-unknown mechanisms that link HSP27 to ANT1 expression appears to be active in cardiomyocytes.





**Figure S1. Phosphorylation of HSP27 and NFκB.** Phosphorylation of HSP27 and NFkB. Graphs show the quantification of HSP27 (A), p85HSP27 protein levels (B), the ratio of pHSP27/HSP27 (C), NFκB/p65 (D), pNFκB/p65 protein levels (E), the ratio of pNFκB/NFκB (F), and ANT1 protein (G) in normoxic (white bars) and hypoxic (blue bars) WT and ANT1-TG cardiomyocytes. The protein level in normoxic WT cardiomyocytes was set to 100% (WTcontrol). n = 5-6, * p < 0.05; ** p < 0.01; *** *p* < 0.001 vs. WTcontrol or vs. indicated conditions; ^##^ *p* < 0.01; ^###^ *p* < 0.001 vs. normoxic ANT1-TG controls.
