# Supplementary figures and images for: Adenine Nucleotide Translocase 1 Expression Is Coupled to the HSP27-Mediated TLR4 Signaling in Cardiomyocytes
Source: Cells. 2019 Dec 6;8(12):1588. doi: 10.3390/cells8121588 (PMC6952976; doi:10.3390/cells8121588)

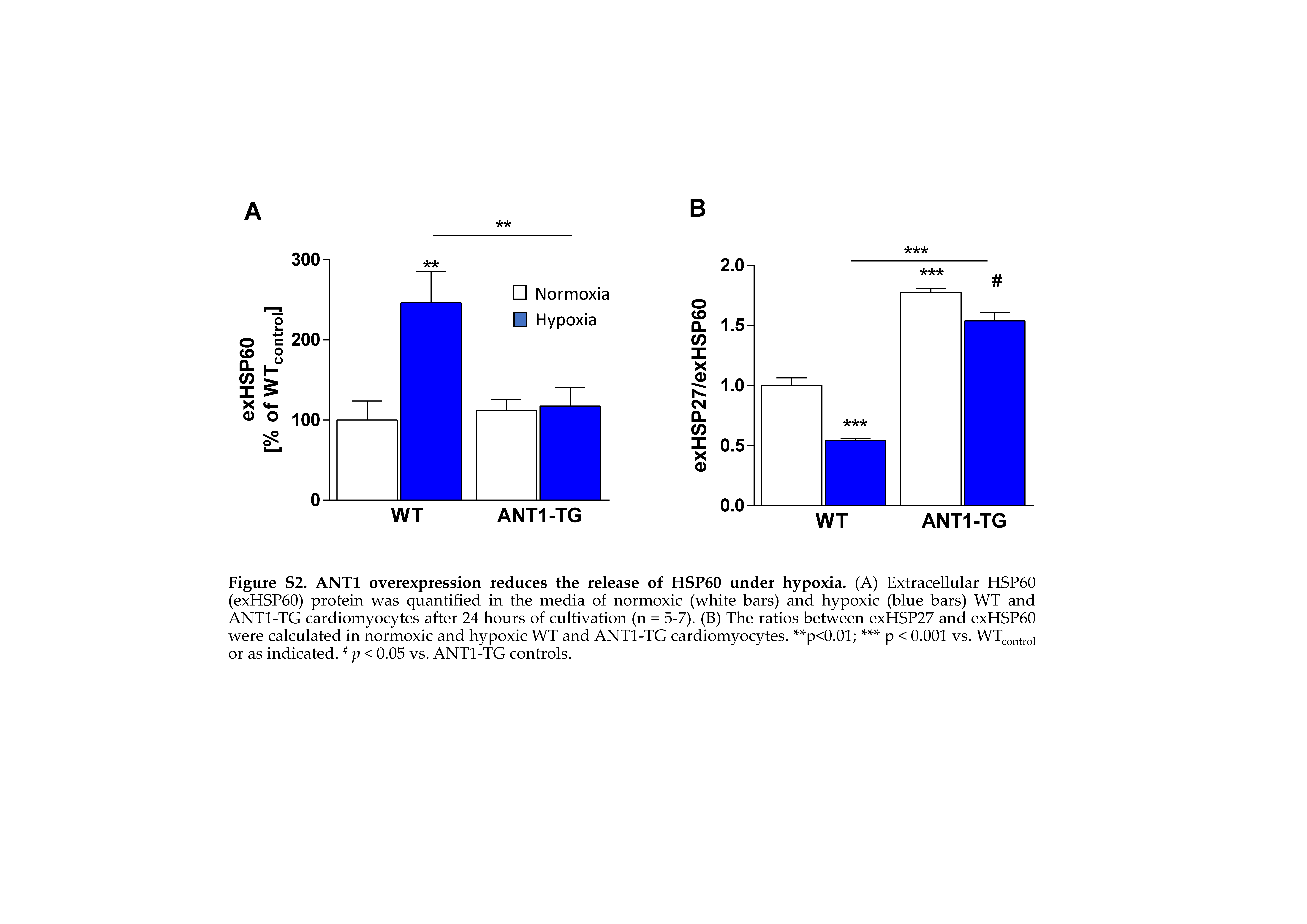

Supplement: Supplementary file 1 [file cells-08-01588-s001.zip › Supplemented files/supplemented Figure S2.tif]
